# Food insecurity and low access to high-quality food for preconception women in Nepal: the importance of household relationships

**DOI:** 10.1017/S1368980020000579

**Published:** 2020-10

**Authors:** Nadia Diamond-Smith, Jacqueline Shieh, Mahesh Puri, Sheri Weiser

**Affiliations:** 1 Department of Epidemiology and Biostatistics, Institute for Global Health Sciences, University of California, San Francisco, CA 94158, USA; 2 Institute for Global Health Sciences, University of California, San Francisco, CA, USA; 3 Center for Research on Environment, Health and Population, Kathmandu, Nepal; 4 Department of Medicine, University of California, San Francisco, CA, USA

**Keywords:** Preconception, Nutrition, South Asia, Household dynamics, Food security

## Abstract

**Objective:**

Women in South Asia, including Nepal, have some of the poorest nutritional indicators globally, leading to poor maternal and child health outcomes. Nepal also suffers from high levels of household food insecurity, and newly married women are at high risk. Intra-household relationships may mediate the relationship between food insecurity and women’s nutrition in Nepal for newly married women. Our aim is to understand how newly married, preconception, women’s food consumption changes when she enters her husband’s home, compared with her natal home. We also explore whether relationship quality with husbands and mothers-in-law mediates the association between food insecurity and eating less high-quality food, using structural equation modelling.

**Design:**

Cross-sectional survey data.

**Setting:**

Rural Nepal in 2018.

**Participants:**

Data were collected from 200 newly married, preconception women.

**Results:**

Women had poor diet quality, and most ate fewer high-quality foods important for pregnancy in their marital, compared with natal, home. Higher quality relationships with mothers-in-laws mediated the association between food insecurity and a woman eating fewer high-quality foods in her marital, compared with natal, home. Relationship quality with husbands was not associated with changes in food consumption.

**Conclusions:**

Preconception, newly married women in Nepal are eating less high-quality foods important for women’s health during the preconception period – a key period for avoiding adverse maternal and infant health outcomes. Relationships with mothers-in-law are key to women’s access to high-quality food, suggesting that interventions aiming to improve maternal and child nutrition should target all household members.

Globally, women in South Asia have the lowest BMI and therefore poorest nutritional outcomes compared with women in other areas of the world^(1,2)^. Women’s poor nutrition and health contribute to high rates of child stunting in South Asia^(1,2)^. Evidence on food allocation suggests that women in South Asia, including in Nepal, receive less food, and lower quality food, than their male counterparts^(3–5)^. This is also true for pregnant women, although some evidence suggests there has been limited improvement in recent years^(3)^. Women in South Asia have lower status and levels of empowerment in their households and communities compared with women in other parts of the world, leaving their nutritional status and health undervalued^(6)^.

Harris-Fry *et al.* ^(7)^ recently published a systematic review of the drivers at the household and community level of unequal food distribution in South Asia. Harris-Fry *et al.* ^(7)^ describe domains that impact intra-household food allocation, including (but not limited to) cultural practices, relative household-level status, bargaining power within the household and food as a means of strengthening relationships. Related to cultural practices and relative household-level status, it is common in South Asia for women to co-reside with their husband’s parents (and possibly other brothers and sisters) and co-residing, in turn, has been found to be a risk factor for low BMI^(8,9)^. Newly married women, who have just moved from their natal (parent’s) home to their new marital home, are at the lowest status in their households and often receive less food than other household members^(9,10)^. In much of South Asia, there is a tradition of the youngest daughter-in-law (most often the newest daughter-in-law) serving the rest of the family and eating last, which has been found to lead to lower consumption of food, and especially of higher quality foods^(4,11)^.

Women’s relationships with other household members are key to understanding their bargaining power and how food is associated with relationship building^(7)^. Harris-Fry *et al.*. specifically mention that young daughter-in-law’s bargaining power may be increased by factors such as high dowry (payment to the grooms family from the brides’ family at the time of marriage). Additionally, Harris-Fry *et al.*. discusses the evidence that the provision of food or certain types of food is a means of showing love; unfortunately, the few studies on this topic in South Asia are over 35 years old^(7)^.

Food security, as defined by the USAID, is ‘when all people at all times have both physical and economic access to sufficient food to meet their dietary needs for a productive and healthy life’ and is usually measured by a validated scale at the household level^(12)^. Gittlesohn and colleagues developed a framework to operationalise household food security in rural Nepal, which highlights the role of intra-household food allocation and related behaviours in understanding how food insecurity might impact individual nutritional intake and health^(13)^. Some studies in South Asia found that unequal distribution of foods is associated with food insecurity, but the data are mixed. For example, in Nepal, this relationship differed by socio-cultural and geographical population^(7,9)^. In India, authors found that the youngest daughters-in-law were most at risk of additional disadvantage in times of food insecurity^(14)^.

Poor preconception nutrition is associated with many adverse outcomes including miscarriage, preeclampsia, low birth weight, small for gestational age, stillbirth, neonatal death and autism^(15)^. Additionally, about half of women in Nepal are pregnant within the first year of marriage, and thus, nutrition in early marriage affects nutrition in pregnancy, a key indicator for maternal and newborn health^(16)^. Despite evidence that youngest daughters-in-law, and especially recently married daughters-in-law, face unequal intra-household allocation of food and are the most at risk of receiving inadequate and poor quality diets, we do not know how this differs from their access to food in their natal homes. We also do not know what other factors are associated with receiving less food, such as the strength and positive nature of the interpersonal relationship between the newly married woman and her husband and mother-in-law is (hereby referred to as ‘relationship quality’). Relationship quality has been found to be important and associated with health outcomes in both qualitative and quantitative research in South Asia^(17–19)^.

In fact, little is known in general about newly married women and their transition into their marital home, especially in comparison with their natal home. We also have little quantitative evidence to understand whether household food security interacts with other household factors to impact new daughter-in-law’s food consumption. We hypothesise that newly married women, especially those living in food-insecure households, will receive less food, especially high-quality food, compared with their natal home. However, we expect this relationship to be mediated by the quality of relationships between the newly married woman and her husband and/or other household members. In this paper, we explore food consumption for newly married women in Nepal, compared with food consumption in their natal homes. Next, we look at the association between household food insecurity and women eating less of high-quality foods in their marital compared with natal home. Finally, we explore if this relationship is mediated by relationship quality with husbands and relationship quality with mothers-in-laws.

## Methods

### Study setting and population

A survey was conducted with 200 newly married women in Nawalparasi district of Nepal, on the border with the Indian state of Uttar Pradesh in February–March 2018. This sample size was estimated to measure a significant difference in stunting of 1·5 between food-secure and food-insecure households (power = 0·80, *α* = 0·05). Nawalparasi district was chosen because it has lower levels of women’s empowerment compared with other districts in Nepal and we were interested in the most disadvantaged women in terms of women’s status^(20)^. Women were eligible if they had been married within the last 4 months, were 18–25 years old and were currently co-residing with their mother-in-law. Newly married women were identified by conducting a mapping of households in one rural municipality (Pali Nandan) and one urban municipality (Sunwal) with the help of community leaders, such as female community health volunteers, teachers, health workers and religious leaders. These municipalities were selected because we believed they were representative of urban and rural municipalities (and therefore women) in this district. A total 302 eligible women (160 women in Sunwal and 142 in Pali Nandan) were identified in the study areas, and 200 women (100 in each area) were selected randomly. Two women were not allowed to participate in the study by their family members and were replaced by the nearest eligible women in the same area. The study size was selected to be able to measure a difference in child stunting by household food insecurity status.

Written informed consent was obtained with women before any interviews were conducted. Interviews were conducted by four trained female Nepali researchers in a private location of the respondent’s choice, usually inside the home or in a nearby field. Interviews with the majority of women were conducted in their local language, Awadhi. Interviews were conducted face-to-face, and data were entered on computer-assisted personal interviews using tablets and KoBo Toolbox version Va.14.0a. KoBo Toolbox is a web-based data system application developed by the Harvard Humanitarian Initiative (https://www.kobotoolbox.org). All data were collected from the newly married women themselves, including the household level items.

## Measures

### Dietary diversity

All data were self-reported (for this and all measures discussed below). We asked fourteen questions about women’s intake of various high-quality food groups (Table 2) over the past month, with the response options of: never, less than once a month, once or twice a month, once or twice a week, most days (3–6 d) or every day. Thus, each item could receive a score ranging from 1 to 6. We modified the module list-based questionnaire from the Minimum Dietary Diversity for Women questionnaire to have food examples relevant and culturally appropriate for the diets of people in this particular part of Nepal^(21)^. These categories had been used by our Nepali research partners. Only high-quality foods are reported in this paper. High-quality foods included: grains, vitamin A-rich foods (e.g. pumpkin, carrots and squash), tubers and roots, dark green and leafy vegetables, fibre-rich fruits (e.g. mango, papaya and banana), other fruits and vegetables, organ meats, meats, eggs, fish or shellfish, legumes and pulses, milk and milk products.

**Table 1:** Women’s demographic characteristics

	*n*		%
Age (years)			
18–19	75		37·5
20–21	68		34
22–23	38		19
24–25	19		9·5
Years of formal education			
No school	8		4
2–4	9		4·5
5–9	71		35·5
10–14	98		49
15 or more	14		7
Has done paid work in the last year	52		26
Religion			
Hindu	172		86
Buddhist	9		4·5
Muslim	17		8·5
Bon/Christian	2		1
Caste			
Brahmin, Chhetri	41		20·5
Janajati, Terai Janjati, Newar	106		53
Other	53		26·5
Spouse works abroad			
No	94		47
Yes, currently	10		5
Yes, in the past	54		27
Yes, in the future	42		21
Number of household members			
3–4	51		25·5
5–6	99		49·5
7 or more	50		25
Lives with			
Husband	186		93
Mother-in-law	199		99·5
Household food insecurity access prevalence			
Food secure	93		46·5
Mildly food insecure	36		18
Moderately food insecure	44		22
Severely food insecure	27		13·5
	Mean	sd	Range
Household food insecurity access scale (HFIAS)	3·000	3·7	0–13

**Table 2:** Food consumption: comparing natal and marital home

Good food groups	Current household (range: 0 (never)–5 (daily))		Natal home					
			More		Same		Less	
	Mean	sd	*n*	%	*n*	%	*n*	%
Roti/chapati, bread, rice, noodle or other foods made from grain (not fried)	4·79	0·57	3	1·51	180	90·5	16	8·04
Pumpkin, carrots, squash or sweet potato that are yellow or orange inside	2·33	1·46	12	7·55	114	71·7	33	20·8
White potato, yams or other foods made from roots	4·69	0·57	8	4·00	180	90·0	12	6·00
Any dark green, leafy vegetables	3·39	1·18	21	11·1	121	63·7	48	25·3
Ripe mango, papaya or banana	2·26	1·46	16	10·1	105	66·5	37	23·4
Dried fruits	2·09	1·57	14	9·15	109	71·2	30	19·6
Any other fruits or vegetables	3·30	0·99	13	6·57	152	76·8	33	16·7
Liver, kidney, heart or other organs of animals	0·61	1·11	10	18·9	30	56·6	13	24·5
Any meat such as buffalo, meat, pork, lamb, chicken, goat or duck	2·64	1·26	26	15·0	93	53·8	54	31·2
Eggs	2·51	1·38	31	18·7	91	54·8	44	26·5
Fresh or dried fish or shellfish	1·54	1·32	19	15·1	67	53·2	40	31·8
Any food made from beans, peas, lentils or nuts	4·46	0·86	15	7·54	166	83·4	18	9·05
Cheese, yogurt or other milk products	1·64	1·53	20	16·0	76	60·8	29	23·2
Milk (tinned, powdered or fresh animal)	2·17	1·84	25	18·7	59	44·0	50	37·3
			Range					
Number of good food groups consumed less of	2·285	2·61	0–14					

### Difference in diet quality

To measure the difference between food consumption in the natal and marital homes, we added an extra question after each food group item in the dietary diversity scale above asking if the woman ate this food group more, about the same, or less than in her natal home (in terms of frequency and quantity). From this, we created a continuous variable about how many high-quality foods the women reported that they ate less in their marital home compared with their natal home. This results in a range of scores from 0 to 14, with higher numbers meaning that women were eating less of more high-quality foods in their husbands’ home compared with their natal home.

### Household food security

Household-level food security was measured using the Household Food Insecurity Access Scale, which was originally validated in Africa through a rigorous process and has been widely used globally^(22)^. Household Food Insecurity Access Scale has also been validated (*α* of 0·75), adapted and used in Nepal, including in the National Demographic and Health Survey (Table 2)^(3,23–27)^.

### Couples relationship quality

Relatively few studies have measured relationship quality quantitatively in the developing world. If measured at all, a simple happiness or satisfaction question is used. Couples relationship quality was measured using a multi-dimensional scale that included four subscales: commitment, dyadic trust, dyadic satisfaction and constructive communication (Table 3)^(28)^. A detailed discussion of these subscales can be found in John *et al*., with references to the original studies, mostly in developed regions, where each scale was validated^(29)^. The combined multi-dimensional scale has previously been used in multiple African countries and has not yet been validated in South Asia. One validation study in Ghana found that only three of these subscales were necessary and thus we also dropped one, the constructive communication scale. This led to a final scale with twenty-one items. There were four response categories for each item: not at all, somewhat, quite and extremely. This resulted in a range from 0 to 3 for each item. A summative scale was created, with high numbers indicating better relationship quality (range 0–63).

**Table 3:** Relationship quality with husband and mother-in-law

	Husband relationship quality			Mother-in-law relationship quality		
	Mean	sd	Range	Mean	sd	Range
Commitment scale*						
I expect my love for my current husband to last for the rest of my life	2·73	0·66	0–3			
I can’t imagine ending my relationship with my current husband	2·72	0·65	0–3			
I view my relationship with my current husband as permanent	2·74	0·61	0–3			
I am committed to maintaining my relationship with my current husband	2·70	0·60	0–3			
I have confidence in the stability of my relationship with my current husband	2·65	0·63	0–3			
Overall	13·5	2·77	0–15			
Trust scale*						
My husband is primarily interested in his own welfare	2·16	0·72	0–3			
There are times when my husband cannot be trusted	2·00	0·78	0–3			
My husband is perfectly honest and truthful with me	2·34	0·65	0–3			
I feel I can trust my husband/mother-in-law completely	2·35	0·62	0–3	2·21	0·57	0–3
My husband is truly sincere in his promises	2·32	0·65	0–3			
I feel that my husband/mother-in-law does not show me enough consideration	2·23	0·68	0–3	2·03	0·71	0–3
My husband/mother-in-law treats me fairly and justly	2·32	0·64	0–3	2·19	0·60	0–3
I feel that my husband/mother-in-law can be counted on to help me	2·39	0·63	0–3	2·20	0·60	0–3
Overall	18·1	4·14	0–24			
Satisfaction scale*						
How often have you discuss or have you considered divorce, separation or termination your relationship?	2·95	0·29	0–3			
How often do you or your husband leave the house after a fight?	2·99	0·12	0–3			
In general, how often do you think that things between you and your husband/mother-in-law are going well?	2·39	0·82	0–3	2·18	0·61	0–3
Do you confide in your husband/mother-in-law?	2·39	0·078	0–3	2·14	0·58	0–3
Do you ever regret that you are married?	2·85	0·54	0–3			
How often do you and your husband quarrel?	2·87	0·48	0–3			
How often do you and your husband ‘get on each other’s nerves?’	2·09	0·67	0–3			
Please rate how happy you are in your relationship	2·44	0·62	0–3	1·47	0·56	0–2
Overall	20·9	3·08	0–24			
Total relationship quality score (three scales combined)*	52·6	8·79	0–63	14·4	3·53	0–20

* Larger number indicates higher relationship quality.

### Mother-in-law relationship quality

Relationship quality with other key household members is under explored, especially quantitatively. There is no known validated (or unvalidated) scale to measure mother-in-law/daughter-in-law relationship quality. Thus, we asked respondents a set of seven questions adapted from the couples’ relationship quality scale described above (Table 3) from which a summative scale was created, with high numbers indicating good relationship quality (range 0–21).

### Socio-demographics

We control for the usual socio-demographics, including age (in years), religion (Hindu *v*. other), caste (higher *v*. lower castes)* and education (in years). To measure household wealth, we used a wealth index and from that created a wealth quintile. We also included a variable for whether the woman was the only daughter-in-law in the household or there were other daughter-in-laws also living in her household, which is a marker of women’s status as younger daughters-in-law have been shown to have the lowest household status (this variable was co-linear with household size; thus, household size was not included)^(30)^. Given the focus on the marriage transition, we also include some marriage-related variables. We include whether the woman had a love or arranged marriage and whether the woman was related to her husband before marriage, both of which have been shown to be associated with various health and social outcomes^(31,32)^. We include a variable for how satisfied the in-laws were with the dowry given (from the woman’s perspective). We also include a variable for how long they had been married at the time of data collection (range was 1–106 d).

## Analysis

We first describe women’s reported dietary diversity by food category, and whether this is more or less than she reported eating in her natal home. Next, we describe women’s reports of relationship quality with husbands and relationship quality with their mothers-in-law.

We conducted mediation analyses for two different models using generalised structural equation modelling. Model A included mother-in-law relationship quality as the potential mediator, while Model B included husband relationship quality as the potential mediator. Generalised structural equation modelling was chosen over structural equation modelling to allow for the use of count responses and to fit negative binomial models. Bootstrapping (*n* 1000) was used to estimate the indirect effects^(33)^ or the extent by which the effect of household food insecurity on the number of high-quality food groups’ participants consumed less of is mediated by mother-in-law relationship quality and/or husband relationship quality. While the causal step method^(34)^ and Sobel test^(35)^ are commonly used to measure indirect effects, we chose to use bootstrapping because it is a non-parametric, bias-corrected test for variables that are not normally distributed and therefore more reliable for our data^(33)^. To calculate the mediation effect size, the mediation ratio was used as an estimate of the proportion of total effect mediated^(36)^. We controlled for socio-demographic variables discussed above.

## Results

All respondents were between 18 and 25 years, with over 70 % being 18–21 years (Table 1). About half had 10–14 years of schooling, with under 10 % having <4 years and 7 % over 12 years (more than high school). About a quarter (26 %) had done paid work in the last year. The majority (86 %) were Hindu. Only 5 % of husbands were currently working abroad, with 21 % planning to in the future and 27 % having done so in the past. Most (56 %) lived in households with 5–6 people, and most (93 %) lived with their husband. Just under half (46·5 %) of household were food-secure, with 18, 22 and 13·5 % being mildly, moderately and severely food-insecure, respectively.

A large percentage of respondents reported frequently eating non-fried foods made from grain, foods made from root and any food made from beans, peas, lentils or nuts (Table 2). Consumption of any dark green, leafy vegetables or orange/yellow vegetable was low, with women, on average reporting eating these items about once or twice a week and once or twice a month, respectively. Fruit consumption was also low, with women reporting eating orange fruits (ripe mango, papaya or banana) and dried fruits on average about once or twice a month and other fruits and vegetables on average about once or twice a week. Meat and dairy protein consumption was also low, with women reporting eating these about once or twice a month or less than once a month.

Respondents mostly reported eating the same amount (frequency and quantity) of food in their husbands’ home compared with their natal home. However, where there was a discrepancy, women more frequently reported receiving a lower quantity of higher quality foods, including 31·8 % eating less fresh or dried fish or shellfish, 31·2 % eating less of any meat such as buffalo, meat, pork, lamb, chicken, goat or duck, 26·5 % eating less eggs, 25·3 % eating less green leafy vegetables, 24·5 % eating less organs meat, 23·4 % eating less ripe mango/papaya or banana and 23·2 % eating less yogurt, or other milk products, compared with their natal home. Women consumed a mean of 2·3 fewer high-quality foods in their marital home compared with natal home (range 0–12).

Table 3 shows the questions asked about the relationship between the woman and her husband, and the woman and her mother-in-law. Overall, women reported fairly high scores with husbands, with a mean score of 13·5/15 on commitment, 18·1/24 on trust and 20·9/24 on satisfaction. Where questions with mothers-in-law aligned with questions about husbands, mothers-in-law received lower scores, most notably on the question about how happy they are in their relationship overall. Please note that the scores have slightly different ranges.

### Model A: Mediation of mother-in-law relationship quality

**Fig. 1:**
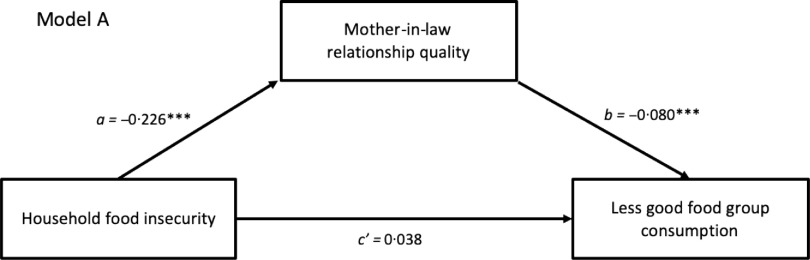
Adjusted generalised structural equation model for the mediation of mother-in-law relationship quality. ***P < 0·01, **P < 0·05, *P < 0·1

The total effects of the mother-in-law relationship quality mediation model indicated an insignificant positive association between household food insecurity and the number of food groups women consumed less of (Table 4, Model A). With the addition of mother-in-law relationship quality to the model, this effect was slightly attenuated. However, the indirect (or mediation) effect of mother-in-law relationship quality was statistically significant (*β* *=* 0·018, 95 % CI 0·005, 0·039), suggesting that mother-in-law relationship quality mediates the relationship between household food insecurity and the number of good food groups that participants consumed less of. The mediation ratio was 0·324, meaning approximately 32·4 % of the total effect was mediated by mother-in-law relationship quality (Fig. 1).

**Table 4:** Total, direct and indirect effects of household food insecurity on less good food group consumption mediated by mother-in-law relationship quality or husband relationship quality†

	Model A (mother-in-law relationship quality)			Model B (husband relationship quality)		
	Path coefficient	se	95 % CI	Path coefficient	se	95 % CI
Total effect	0·056*	0·031	–0·004, 0·116	0·057*	0·031	–0·003, 0·117
Direct effect	0·038	0·030	–0·022, 0·097	0·044	0·031	–0·016, 0·104
Indirect effect	0·018**	0·009	0·005, 0·039	0·013	0·008	0·001, 0·033

† CI for indirect effects generated with bias corrected bootstrapping (*n* 1000).

***P < 0·01, **P < 0·05, *P < 0·1.

### Model B: Mediation of husband relationship quality

**Fig. 2:**
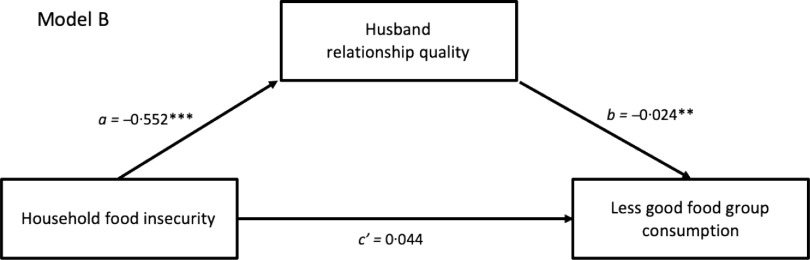
Adjusted generalised structural equation model for the mediation of husband relationship quality. ***P < 0·01, **P < 0·05, *P < 0·1

Similar to the mother-in-law mediation model, the total effects of the husband relationship quality indicated that household food insecurity and the number of food groups that women consumed less of were not significantly statistically correlated (Table 4, Model B). This effect also slightly decreased with the addition of spousal relationship quality to the model. However, the indirect effect of husband relationship quality was not statistically significant (*β* *=* 0·013, 95 % CI 0·001, 0·033), which suggests that the effect of household food insecurity on the number of good food groups women consumed less of is not mediated by husband relationship quality (Fig. 2).

## Discussion

A substantial proportion of young, newly married, preconception women are eating less high-quality food after they are married, compared with when they were living in their parents’ home. Preconception nutrition in itself is important for maternal and newborn health, and given that about half of newly married women are pregnant within 1 year of marriage, this time period quickly rolls into the pregnancy period, where nutrition is key. Promoting the importance of nutrition pre-pregnancy, as well as into pregnancy, is essential and is currently neglected in this setting. One study in Nepal found that 96 % of pregnant women had micronutrient deficiencies and about 18 % were deficient in five or more micronutrients^(37)^. We find that women are eating less Fe-rich foods in particular, which is associated with anaemia. Anaemia rates are very high in Nepal (34 % of all women, 48 % of pregnancy women)^(38,39)^, and if a woman is anaemic when she gets pregnant, it is difficult to replete in such a short-time period. Anaemia in pregnancy is associated with preterm labour, haemorrhage, shock and heart failure for the mother, and for the child with stillbirth, newborn morbidity and mortality, prematurity, and long-term impacts on physical and cognitive development and impairment^(40)^. Household-level socio-cultural practices around order of food consumption and who receives high-quality foods are likely putting preconception women at risk of poor nutrition. There is some evidence that nutrition interventions can address these household behaviours. An intervention in Nepal that tested participatory women’s groups with cash compared with groups with fortified flour transfers found that groups with cash had improved dietary diversity and groups with flour had improved distribution of food resources within households (between women and mothers-in-law specifically)^(41)^. An intervention in Bangladesh with improved vegetables and aquaculture technology found that when the programme was rolled out in groups of women as opposed to individual women, it made more of an impact on household gender relationships^(42)^.

We also find that the association between household-level food insecurity and eating less high-quality food upon moving into the marital home is mediated by having a better relationship with mothers-in-law. However, relationship quality with husbands does not mediate the relationship between food insecurity and women’s access to more high-quality food. In this setting of household-level decision-making and the low status of newly married women, it is clear that engaging mothers-in-laws and helping to improve relationships between newly married women and their mothers-in-law may be key for improving women’s health in this critical time period. A few known interventions in South Asia have worked at the household level, including one in India between mothers-in-law and daughters-in-law to reduce domestic violence^(43)^. Another intervention in India focused on improving the relationship between adolescent girls and their parents to reduce school dropout^(44)^. Such an intervention could be adapted to newly married women and their mothers-in-law, with a focus on nutrition and other health-related behaviours.

While our study has several strengths, including our novel study population and measuring an unexplored area of nutrition key to preconception health – the transition from natal to husbands’ home, it also has several limitations. This study is cross-sectional and with a relatively small sample. All data were collected in a short time window and therefore are only reflective of food consumption in March and April; however, there is seasonality in consumption of foods, including important fruits, in this setting, which we cannot capture. Additionally, all data are self-reported and may suffer from recall bias, especially for questions about food consumption in the natal home. However, all women had been married under 4 months, so recall bias should not be too large of an issue. Although part of the goal was understanding the relationships and behaviour very early in marriage, these relationships are likely to change after the first few months of marriage and therefore are not reflective of relationships and behaviours later in marriage.

Preconception and pregnancy nutrition need more attention, especially in South Asia where women have some of the poorest nutritional indicators globally. Programmes and interventions should focus resources on newly married women and their households, working specifically to improve household relationships as a means of improving newly married women’s access to high-quality foods and ultimately their health and the health of their future children.

## Footnotes

* Caste is a measure of social standing in Hindu society
